# Vitamin C and Immune Function

**DOI:** 10.3390/nu9111211

**Published:** 2017-11-03

**Authors:** Anitra C. Carr, Silvia Maggini

**Affiliations:** 1 Department of Pathology, University of Otago, Christchurch, P.O. Box 4345, Christchurch 8140, New Zealand; 2 Bayer Consumer Care Ltd., Peter-Merian-Strasse 84, 4002 Basel, Switzerland; silvia.maggini@bayer.com

**Keywords:** ascorbate, ascorbic acid, immunity, immune system, neutrophil function, microbial killing, lymphocytes, infection, vitamin C

## Abstract

Vitamin C is an essential micronutrient for humans, with pleiotropic functions related to its ability to donate electrons. It is a potent antioxidant and a cofactor for a family of biosynthetic and gene regulatory enzymes. Vitamin C contributes to immune defense by supporting various cellular functions of both the innate and adaptive immune system. Vitamin C supports epithelial barrier function against pathogens and promotes the oxidant scavenging activity of the skin, thereby potentially protecting against environmental oxidative stress. Vitamin C accumulates in phagocytic cells, such as neutrophils, and can enhance chemotaxis, phagocytosis, generation of reactive oxygen species, and ultimately microbial killing. It is also needed for apoptosis and clearance of the spent neutrophils from sites of infection by macrophages, thereby decreasing necrosis/NETosis and potential tissue damage. The role of vitamin C in lymphocytes is less clear, but it has been shown to enhance differentiation and proliferation of B- and T-cells, likely due to its gene regulating effects. Vitamin C deficiency results in impaired immunity and higher susceptibility to infections. In turn, infections significantly impact on vitamin C levels due to enhanced inflammation and metabolic requirements. Furthermore, supplementation with vitamin C appears to be able to both prevent and treat respiratory and systemic infections. Prophylactic prevention of infection requires dietary vitamin C intakes that provide at least adequate, if not saturating plasma levels (i.e., 100–200 mg/day), which optimize cell and tissue levels. In contrast, treatment of established infections requires significantly higher (gram) doses of the vitamin to compensate for the increased inflammatory response and metabolic demand.

## 1. Introduction

The immune system is a multifaceted and sophisticated network of specialized organs, tissues, cells, proteins, and chemicals, which has evolved in order to protect the host from a range of pathogens, such as bacteria, viruses, fungi, and parasites, as well as cancer cells [1]. It can be divided into epithelial barriers, and cellular and humoral constituents of either innate (non-specific) and acquired (specific) immunity [1]. These constituents interact in multiple and highly complex ways. More than half a century of research has shown vitamin C to be a crucial player in various aspects of the immune system, particularly immune cell function [2,3].

Vitamin C is an essential nutrient which cannot be synthesized by humans due to loss of a key enzyme in the biosynthetic pathway [4,5]. Severe vitamin C deficiency results in the potentially fatal disease scurvy [6]. Scurvy is characterized by weakening of collagenous structures, resulting in poor wound healing, and impaired immunity. Individuals with scurvy are highly susceptible to potentially fatal infections such as pneumonia [7]. In turn, infections can significantly impact on vitamin C levels due to enhanced inflammation and metabolic requirements. Early on, it was noted that scurvy often followed infectious epidemics in populations [7], and cases of scurvy have been reported following respiratory infection [8]. This is particularly apparent for individuals who are already malnourished.

Although the amount of vitamin C required to prevent scurvy is relatively low (i.e., ~10 mg/day) [9], the recommended dietary intakes for vitamin C are up to one hundred-fold higher than that for many other vitamins [10]. A diet that supplies 100–200 mg/day of vitamin C provides adequate to saturating plasma concentrations in healthy individuals and should cover general requirements for the reduction of chronic disease risk [11,12]. Due to the low storage capacity of the body for the water-soluble vitamin, a regular and adequate intake is required to prevent hypovitaminosis C. Epidemiological studies have indicated that hypovitaminosis C (plasma vitamin C < 23 μmol/L) is relatively common in Western populations, and vitamin C deficiency (<11 μmol/L) is the fourth leading nutrient deficiency in the United States [13,14]. There are several reasons why vitamin C dietary recommendations are not met, even in countries where food availability and supply would be expected to be sufficient. These include poor dietary habits, life-stages and/or lifestyles either limiting intakes or increasing micronutrient requirements (e.g., smoking and alcohol or drug abuse), various diseases, exposure to pollutants and smoke (both active and passive), and economic reasons (poor socioeconomic status and limited access to nutritious food) [15,16]. Even otherwise ‘healthy’ individuals in industrialized countries can be at risk due to lifestyle-related factors, such as those on a diet or eating an unbalanced diet, and people facing periods of excessive physical or psychological stress [15,16].

**Figure 1:**
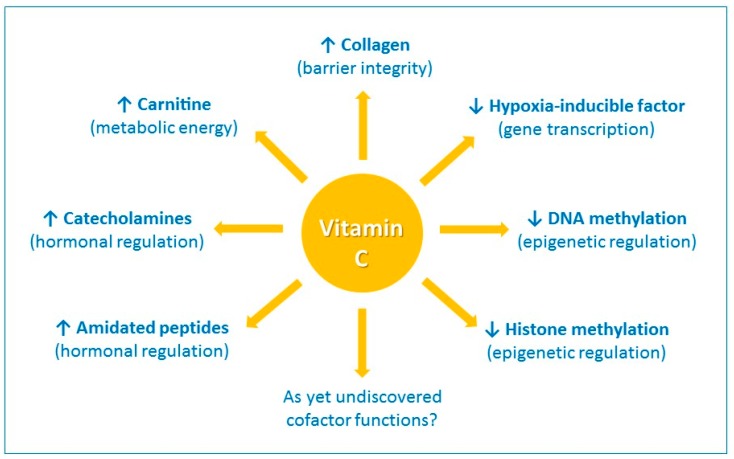
The enzyme cofactor activities of vitamin C. Vitamin C is a cofactor of a family of biosynthetic and gene regulatory monooxygenase and dioxygenase enzymes. These enzymes are involved in the synthesis of collagen, carnitine, catecholamine hormones, e.g., norepinephrine, and amidated peptide hormones, e.g., vasopressin. These enzymes also hydroxylate transcription factors, e.g., hypoxia-inducible factor 1α, and methylated DNA and histones, thus playing a role in gene transcription and epigenetic regulation. ↑ indicates an increase and ↓ indicates a decrease.

Vitamin C has a number of activities that could conceivably contribute to its immune-modulating effects. It is a highly effective antioxidant, due to its ability to readily donate electrons, thus protecting important biomolecules (proteins, lipids, carbohydrates, and nucleic acids) from damage by oxidants generated during normal cell metabolism and through exposure to toxins and pollutants (e.g., cigarette smoke) [17]. Vitamin C is also a cofactor for a family of biosynthetic and gene regulatory monooxygenase and dioxygenase enzymes [18,19]. The vitamin has long been known as a cofactor for the lysyl and prolyl hydroxylases required for stabilization of the tertiary structure of collagen, and is a cofactor for the two hydroxylases involved in carnitine biosynthesis, a molecule required for transport of fatty acids into mitochondria for generation of metabolic energy (Figure 1) [19].

Vitamin C is also a cofactor for the hydroxylase enzymes involved in the synthesis of catecholamine hormones, e.g., norepinephrine, and amidated peptide hormones e.g., vasopressin, which are central to the cardiovascular response to severe infection [20]. Furthermore, research over the past 15 years or so has uncovered new roles for vitamin C in the regulation of gene transcription and cell signaling pathways through regulation of transcription factor activity and epigenetic marks (Figure 1) [21,22]. For example, the asparagyl and prolyl hydroylases required for the downregulation of the pleiotropic transcription factor hypoxia-inducible factor-1α (HIF-1α) utilize vitamin C as a cofactor [21]. Recent research has also indicated an important role for vitamin C in regulation of DNA and histone methylation by acting as a cofactor for enzymes which hydoxylate these epigenetic marks [22].

Our review explores the various roles of vitamin C in the immune system, including barrier integrity and leukocyte function, and discusses potential mechanisms of action. We discuss the relevance of the immune-modulating effects of vitamin C in the context of infections and conditions leading to vitamin C insufficiency.

## 2. Barrier Integrity and Wound Healing

The skin has numerous essential functions, the primary of which is to act as a barrier against external insults, including pathogens. The epidermal layer is highly cellular, comprising primarily keratinocytes, whilst the dermal layer comprises fibroblasts which secrete collagen fibers, the major component of the dermis [23]. Skin contains millimolar concentrations of vitamin C, with higher levels found in the epidermis than the dermis [24,25,26]. Vitamin C is actively accumulated into the epidermal and dermal cells via the two sodium-dependent vitamin C transporter (SVCT) isoforms 1 and 2 [27], suggesting that the vitamin has crucial functions within the skin. Clues to the role of vitamin C in the skin come from the symptoms of the vitamin C deficiency disease scurvy, which is characterized by bleeding gums, bruising, and impaired wound healing [28,29]. These symptoms are thought to be a result of the role of vitamin C as a co-factor for the prolyl and lysyl hydroxylase enzymes that stabilize the tertiary structure of collagen (Table 1) [30]. Further research has shown that vitamin C can also increase collagen gene expression in fibroblasts [31,32,33,34,35].

Vitamin C intervention studies in humans (using both dietary and gram doses of vitamin C) have shown enhanced vitamin C uptake into skin cells [26,36] and enhanced oxidant scavenging activity of the skin [36,37]. The elevated antioxidant status of the skin following vitamin C supplementation could potentially protect against oxidative stress induced by environmental pollutants [38,39]. The antioxidant effects of vitamin C are likely to be enhanced in combination with vitamin E [40,102].

Cell culture and preclinical studies have indicated that vitamin C can enhance epithelial barrier functions via a number of different mechanisms. Vitamin C supplementation of keratinocytes in culture enhances differentiation and barrier function via modulating signaling and biosynthetic pathways, with resultant elevations in barrier lipid synthesis [41,42,43,44,45]. Dysfunctional epithelial barrier function in the lungs of animals with serious infection can be restored by administration of vitamin C [74]. This was attributed to enhanced expression of tight junction proteins and prevention of cytoskeletal rearrangements.

Animal studies using the vitamin C-dependent Gulo knockout mouse indicated that deficiency did not affect the formation of collagen in the skin of unchallenged mice [103]; however, following full thickness excisional wounding there was significantly decreased collagen formation in vitamin C-deficient mice [46]. This finding is in agreement with an earlier study carried out with scorbutic guinea pigs [104]. Thus, vitamin C appears to be particularly essential during wound healing, also decreasing the expression of pro-inflammatory mediators and enhancing the expression of various wound healing mediators [46]. Fibroblast cell culture experiments have also indicated that vitamin C can alter gene expression profiles within dermal fibroblasts, promoting fibroblast proliferation and migration which is essential for tissue remodeling and wound healing [46,47]. Following surgery, patients require relatively high intakes of vitamin C in order to normalize their plasma vitamin C status (e.g., ≥500 mg/day) [105], and administration of antioxidant micronutrients, including vitamin C, to patients with disorders in wound healing can shorten the time to wound closure [48,49,106,107].

Leukocytes, particularly neutrophils and monocyte-derived macrophages, are major players in wound healing [108]. During the initial inflammatory stage, neutrophils migrate to the wound site in order to sterilize it via the release of reactive oxygen species (ROS) and antimicrobial proteins [109]. The neutrophils eventually undergo apoptosis and are cleared by macrophages, resulting in resolution of the inflammatory response. However, in chronic, non-healing wounds, such as those observed in diabetics, the neutrophils persist and instead undergo necrotic cell death which can perpetuate the inflammatory response and hinder wound healing [109,110]. Vitamin C is thought to influence several important aspects of neutrophil function: migration in response to inflammatory mediators (chemotaxis), phagocytosis and killing of microbes, and apoptosis and clearance by macrophages (see below).

## 3. Vitamin C and Leukocyte Function

Leukocytes, such as neutrophils and monocytes, actively accumulate vitamin C against a concentration gradient, resulting in values that are 50- to 100-fold higher than plasma concentrations [111,112,113]. These cells accumulate maximal vitamin C concentrations at dietary intakes of ~100 mg/day [114,115], although other body tissues likely require higher intakes for saturation [116,117]. Neutrophils accumulate vitamin C via SVCT2 and typically contain intracellular levels of at least 1 mM [111,118]. Following stimulation of their oxidative burst neutrophils can further increase their intracellular concentration of vitamin C through the non-specific uptake of the oxidized form, dehydroascorbate (DHA), via glucose transporters (GLUT) [118,119]. DHA is then rapidly reduced to ascorbate intracellularly, to give levels of about 10 mM [119]. It is believed that the accumulation of such high vitamin C concentrations indicates important functions within these cells.

Accumulation of millimolar concentrations of vitamin C into neutrophils, particularly following activation of their oxidative burst, is thought to protect these cells from oxidative damage [119]. Vitamin C is a potent water-soluble antioxidant that can scavenge numerous reactive oxidants and can also regenerate the important cellular and membrane antioxidants glutathione and vitamin E [120]. Upon phagocytosis or activation with soluble stimulants, vitamin C is depleted from neutrophils in an oxidant-dependent manner [50,51,52,53]. An alteration in the balance between oxidant generation and antioxidant defenses can lead to alterations in multiple signaling pathways, with the pro-inflammatory transcription factor nuclear factor кB (NFкB) playing a central role [121]. Oxidants can activate NFкB, which triggers a signaling cascade leading to continued synthesis of oxidative species and other inflammatory mediators [122,123]. Vitamin C has been shown to attenuate both oxidant generation and NFкB activation in dendritic cells in vitro, and NFкB activation in neutrophils isolated from septic Gulo knockout mice [75,124]. Thiol-containing proteins can be particularly sensitive to redox alterations within cells and are often central to the regulation of redox-related cell signaling pathways [125]. Vitamin C-dependent modulation of thiol-dependent cell signaling and gene expression pathways has been reported in T-cells [126,127].

Thus, vitamin C could modulate immune function through modulation of redox-sensitive cell signaling pathways or by directly protecting important cell structural components. For example, exposure of neutrophils to oxidants can inhibit motility of the cells, which is thought to be due to oxidation of membrane lipids and resultant effects on cell membrane fluidity [63]. Neutrophils contain high levels of polyunsaturated fatty acids in their plasma membranes, and thus improvements in neutrophil motility observed following vitamin C administration (see below) could conceivably be attributed to oxidant scavenging as well as regeneration of vitamin E [120].

### 3.1. Neutrophil Chemotaxis

**Figure 2:**
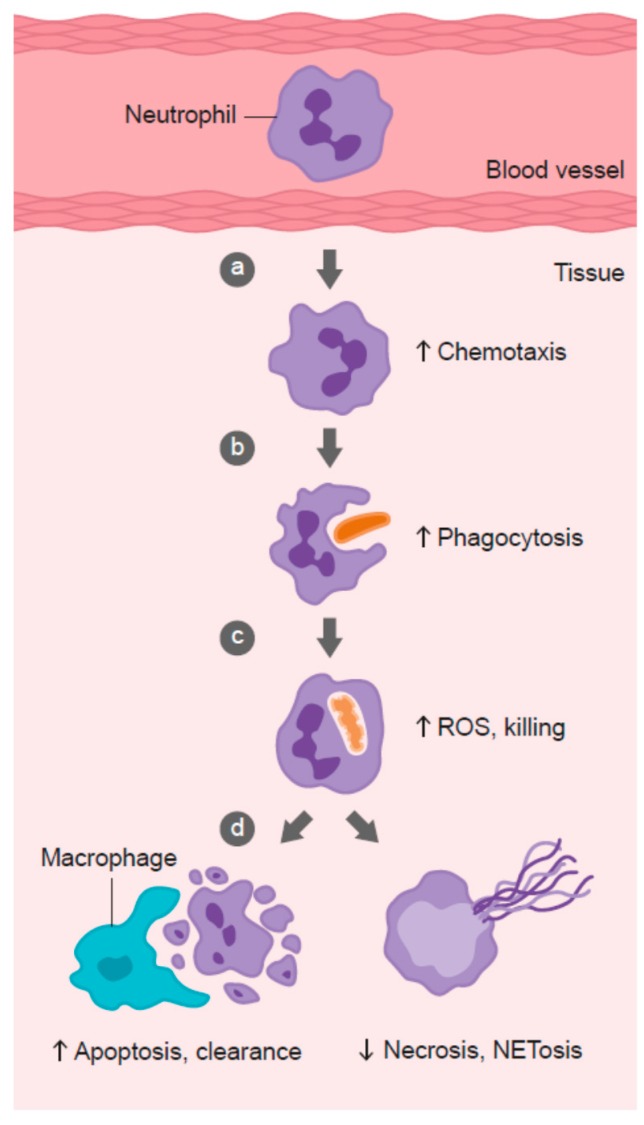
Role of vitamin C in phagocyte function. Vitamin C has been shown to: (**a**) enhance neutrophil migration in response to chemoattractants (chemotaxis), (**b**) enhance engulfment (phagocytosis) of microbes, and (**c**) stimulate reactive oxygen species (ROS) generation and killing of microbes. (**d**) Vitamin C supports caspase-dependent apoptosis, enhancing uptake and clearance by macrophages, and inhibits necrosis, including NETosis, thus supporting resolution of the inflammatory response and attenuating tissue damage.

Neutrophil infiltration into infected tissues is an early step in innate immunity. In response to pathogen- or host-derived inflammatory signals (e.g., *N*-formylmethionyl-leucyl-phenylalanine (fMLP), interleukin (IL)-8, leukotriene B4, and complement component C5a), marginated neutrophils literally swarm to the site of infection [128]. Migration of neutrophils in response to chemical stimuli is termed chemotaxis, while random migration is termed chemokinesis (Figure 2). Neutrophils express more than 30 different chemokine and chemoattractant receptors in order to sense and rapidly respond to tissue damage signals [128]. Early studies carried out in scorbutic guinea pigs indicated impaired leukocyte chemotactic response compared with leukocytes isolated from guinea pigs supplemented with adequate vitamin C in their diet (Table 1) [54,55,56,64]. These findings suggest that vitamin C deficiency may impact on the ability of phagocytes to migrate to sites of infection.

Patients with severe infection exhibit compromised neutrophil chemotactic ability [129,130,131,132]. This neutrophil ‘paralysis’ is believed to be partly due to enhanced levels of anti-inflammatory and immune-suppressive mediators (e.g., IL-4 and IL-10) during the compensatory anti-inflammatory response observed following initial hyper-stimulation of the immune system [133]. However, it is also possible that vitamin C depletion, which is prevalent during severe infection [20], may contribute. Studies in the 1980s and 1990s indicated that patients with recurrent infections had impaired leukocyte chemotaxis, which could be restored in response to supplementation with gram doses of vitamin C [57,58,59,60,65,66,67]. Furthermore, supplementation of neonates with suspected sepsis with 400 mg/day vitamin C dramatically improved neutrophil chemotaxis [134].

Recurrent infections can also result from genetic disorders of neutrophil function, such as chronic granulomatous disease (CGD), an immunodeficiency disease resulting in defective leukocyte generation of ROS [135], and Chediak-Higashi syndrome (CHS), a rare autosomal recessive disorder affecting vesicle trafficking [136]. Although vitamin C administration would not be expected to affect the underlying defects of these genetic disorders, it may support the function of redundant antimicrobial mechanisms in these cells. For example, patients with CGD showed improved leukocyte chemotaxis following supplementation with gram doses of vitamin C administered either enterally or parenterally [137,138,139]. This was associated with decreased infections and clinical improvement [137,138]. A mouse model of CHS showed improved neutrophil chemotaxis following vitamin C supplementation [140], and neutrophils isolated from two children with CHS showed improved chemotaxis following supplementation with 200–500 mg/day vitamin C [141,142], although this effect has not been observed in all cases [140,143]. The vitamin C-dependent enhancement of chemotaxis was thought to be mediated in part via effects on microtubule assembly [144,145], and more recent research has indicated that intracellular vitamin C can stabilize microtubules [146].

Supplementation of healthy volunteers with dietary or gram doses of vitamin C has also been shown to enhance neutrophil chemotactic ability [61,62,63,147]. Johnston et al., proposed that the antihistamine effect of vitamin C correlated with enhanced chemotaxis [61]. In participants who had inadequate vitamin C status (i.e., <50 µM), supplementation with a dietary source of vitamin C (providing ~250 mg/day) resulted in a 20% increase in neutrophil chemotaxis [147]. Furthermore, supplementation of elderly women with 1 g/day vitamin C, in combination with vitamin E, enhanced neutrophil functions, including chemotaxis [148]. Thus, members of the general population may benefit from improved immune cell function through enhanced vitamin C intake, particularly if they have inadequate vitamin C status, which can be more prevalent in the elderly. However, it should be noted that it is not yet certain to what extent improved ex vivo leukocyte chemotaxis translates into improved in vivo immune function.

### 3.2. Phagocytosis and Microbial Killing

Once neutrophils have migrated to the site of infection, they proceed to engulf the invading pathogens (Figure 2). Various intracellular granules are mobilized and fuse with the phagosome, emptying their arsenal of antimicrobial peptides and proteins into the phagosome [149]. Components of the nicotinamide adenine dinucleotide phosphate (NADPH) oxidase assemble in the phagosomal membrane and generate superoxide, the first in a long line of ROS generated by neutrophils to kill pathogens. The enzyme superoxide dismutase converts superoxide to hydrogen peroxide, which can then be utilized to form the oxidant hypochlorous acid via the azurophilic granule enzyme myeloperoxidase [149]. Hypochlorous acid can further react with amines to form secondary oxidants known as chloramines. These various neutrophil-derived oxidants have different reactivities and specificities for biological targets, with protein thiol groups being particularly susceptible.

Neutrophils isolated from scorbutic guinea pigs exhibit a severely impaired ability to kill microbes [54,55,70], and studies have indicated impaired phagocytosis and/or ROS generation in neutrophils from scorbutic compared with ascorbate replete animals [68,69,70]. Generation of ROS by neutrophils from volunteers with inadequate vitamin C status can be enhanced by 20% following supplementation with a dietary source of vitamin C [147], and increases in both phagocytosis and oxidant generation were observed following supplementation of elderly participants with a combination of vitamins C and E [148]. Patients with recurrent infections [57,58,66,67,72], or the genetic conditions CGD or CHS [138,139,141,143,150], have impaired neutrophil bacterial killing and/or phagocytosis, which can be significantly improved following supplementation with gram doses of vitamin C, resulting in long lasting clinical improvement. A couple of studies, however, showed no improvement of ex vivo anti-fungal or anti-bacterial activity in neutrophils isolated from CGD or CHS patients supplemented with vitamin C [140,151]. The reason for these differences is not clear, although it may depend on the baseline vitamin C level of the patients, which is not assessed in most cases. Furthermore, different microbes have variable susceptibility to the oxidative and non-oxidative anti-microbial mechanisms of neutrophils. For example, *Staphylococcus aureus* is susceptible to oxidative mechanisms, whereas other microorganisms are more susceptible to non-oxidative mechanisms [152]. Therefore, the type of microbe used to assess the ex vivo neutrophil functions could influence the findings.

Patients with severe infection (sepsis) exhibit a decreased ability to phagocytose microbes and a diminished ability to generate ROS [153]. Decreased neutrophil phagocytosis was associated with enhanced patient mortality [154]. Interestingly, Stephan et al. [155] observed impaired neutrophil killing activity in critically ill patients prior to acquiring nosocomial infections, suggesting that critical illness itself, without prior infection, can also impair neutrophil function. This resulted in subsequent susceptibility to hospital-acquired infections. Impaired phagocytic and oxidant-generating capacity of leukocytes in patients with severe infection has been attributed to the compensatory anti-inflammatory response, resulting in enhanced levels of immunosuppressive mediators such as IL-10 [133], as well as to the hypoxic conditions of inflammatory sites, which diminishes substrate for ROS generation [156]. Another explanation is the larger numbers of immature neutrophils released from the bone marrow due to increased demands during severe infection. These immature ‘band’ cells have decreased functionality compared with differentiated neutrophils [157]. Thus, conflicting findings in severe infection could be due to variability in the total numbers of underactive immature neutrophils compared with activated fully-differentiated neutrophils [158,159]. Despite displaying an activated basal state, the mature neutrophils from patients with severe infection do not generate ROS to the same extent as healthy neutrophils following ex vivo stimulation [160]. The effect of vitamin C supplementation on phagocytosis, oxidant generation, and microbial killing by leukocytes from septic patients has not yet been explored.

### 3.3. Neutrophil Apoptosis and Clearance

Following microbial phagocytosis and killing, neutrophils undergo a process of programmed cell death called apoptosis [161]. This process facilitates subsequent phagocytosis and clearance of the spent neutrophils from sites of inflammation by macrophages, thus supporting resolution of inflammation and preventing excessive tissue damage (Figure 2). Caspases are key effector enzymes in the apoptotic process, culminating in phosphatidyl serine exposure, thus marking the cells for uptake and clearance by macrophages [162]. Interestingly, caspases are thiol-dependent enzymes, making them very sensitive to inactivation by ROS generated by activated neutrophils [163,164]. Thus, vitamin C may be expected to protect the oxidant-sensitive caspase-dependent apoptotic process following activation of neutrophils. In support of this premise, in vitro studies have shown that loading human neutrophils with vitamin C can enhance *Escherichia coli*-mediated apoptosis of the neutrophils (Table 1) [71]. Peritoneal neutrophils isolated from vitamin C-deficient Gulo mice exhibited attenuated apoptosis [75], and instead underwent necrotic cell death [73]. These vitamin C-deficient neutrophils were not phagocytosed by macrophages in vitro, and persisted at inflammatory loci in vivo [73]. Furthermore, administration of vitamin C to septic animals decreased the numbers of neutrophils in the lungs of these animals [74].

Numerous studies have reported attenuated neutrophil apoptosis in patients with severe infection compared with control participants [165,166,167,168,169,170,171,172]. The delayed apoptosis appears to be related to disease severity and is thought to be associated with enhanced tissue damage observed in patients with sepsis [173,174]. Immature ‘band’ neutrophils released during severe infection were also found to be resistant to apoptosis and had longer life spans [157]. Plasma from septic patients has been found to suppress apoptosis in healthy neutrophils, suggesting that pro-inflammatory cytokines were responsible for the increased in vivo survival of neutrophils during inflammatory conditions [165,174,175,176]. Interestingly, high-dose vitamin C administration has been shown to modulate cytokine levels in patients with cancer [177] and, although this has not yet been assessed in patients with severe infection, could conceivably be another mechanism by which vitamin C may modulate neutrophil function in these patients. To date, only one study has investigated the effect of vitamin C supplementation on neutrophil apoptosis in septic patients [178]. Intravenous supplementation of septic abdominal surgery patients with 450 mg/day vitamin C was found to decrease caspase-3 protein levels and, thus was presumed to have an anti-apoptotic effect on peripheral blood neutrophils. However, caspase activity and apoptosis of the neutrophils following activation was not assessed. Furthermore, circulating neutrophils may not reflect the activation status of neutrophils at inflammatory tissue loci. Clearly, more studies need to be undertaken to tease out the role of vitamin C in neutrophil apoptosis and clearance from inflammatory loci.

### 3.4. Neutrophil Necrosis and NETosis

Neutrophils that fail to undergo apoptosis instead undergo necrotic cell death (Figure 2). The subsequent release of toxic intracellular components, such as proteases, can cause extensive tissue damage [179,180]. One recently discovered form of neutrophil death has been termed NETosis. This results from the release of ‘neutrophil extracellular traps’ (NETs) comprising neutrophil DNA, histones, and enzymes [181]. Although NETs have been proposed to comprise a unique method of microbial killing [182,183], they have also been implicated in tissue damage and organ failure [184,185]. NET-associated histones can act as damage-associated molecular pattern proteins, activating the immune system and causing further damage [186]. Patients with sepsis, or who go on to develop sepsis, have significantly elevated levels of circulating cell-free DNA, which is thought to indicate NET formation [184,187].

Pre-clinical studies in vitamin C-deficient Gulo knockout mice indicated enhanced NETosis in the lungs of septic animals and increased circulating cell-free DNA [75]. The levels of these markers were attenuated in vitamin C sufficient animals or in deficient animals that were administered vitamin C (Table 1). The same investigators showed that in vitro supplementation of human neutrophils with vitamin C attenuated phorbol ester-induced NETosis [75]. Administration of gram doses of vitamin C to septic patients over four days, however, did not appear to decrease circulating cell-free DNA levels [188], although the duration of treatment may have been too short to see a sustained effect. It should be noted that cell-free DNA is not specific for neutrophil-derived DNA, as it may also derive from necrotic tissue; however, the association of neutrophil-specific proteins or enzymes, such as myeloperoxidase, with the DNA can potentially provide an indication of its source [184].

The transcription factor HIF-1α facilitates neutrophil survival at hypoxic loci through delaying apoptosis [189]. Interestingly, vitamin C is a cofactor for the iron-containing dioxygenase enzymes that regulate the levels and activity of HIF-1α [190]. These hydroxylase enzymes downregulate HIF-1α activity by facilitating degradation of constitutively expressed HIF-1α and decreasing binding of transcription coactivators. In vitamin C-deficient Gulo knockout mice, up-regulation of HIF-1α was observed under normoxic conditions, along with attenuated neutrophil apoptosis and clearance by macrophages [73]. HIF-1α has also been proposed as a regulator of NET generation by neutrophils [191], hence providing a potential mechanism by which vitamin C could downregulate NET generation by these cells [75].

### 3.5. Lymphocyte Function

Like phagocytes, B- and T-lymphocytes accumulate vitamin C to high levels via SVCT [192,193]. The role of vitamin C within these cells is less clear, although antioxidant protection has been suggested [194]. In vitro studies have indicated that incubation of vitamin C with lymphocytes promotes proliferation [76,77], resulting in enhanced antibody generation [78], and also provides resistance to various cell death stimuli [195]. Furthermore, vitamin C appears to have an important role in developmental differentiation and maturation of immature T-cells (Table 1) [76,79]. Similar proliferative and differentiation/maturation effects have been observed with mature and immature natural killer cells, respectively [196].

Early studies in guinea pigs showed enhanced mitotic activity of isolated peripheral blood lymphocytes following intraperitoneal vitamin C treatment, and enhanced humoral antibody levels during immunization [82,83,84,85]. Although one human intervention study has reported positive associations between antibody levels (immunoglobulin (Ig)M, (Ig)G, (Ig)A) and vitamin C supplementation [85], another has not [62]. Instead, Anderson and coworkers showed that oral and intravenous supplementation of low gram doses of vitamin C to children with asthma and healthy volunteers enhanced lymphocyte transformation, an ex vivo measure of mitogen-induced proliferation and enlargement of T-lymphocytes (Table 1) [62,63,81]. Administration of vitamin C to elderly people was also shown to enhance ex vivo lymphocyte proliferation [80], a finding confirmed using combinations of vitamin C with vitamins A and/or E [148,197]. Exposure to toxic chemicals can affect lymphocyte function, and both natural killer cell activity and lymphocyte blastogenic responses to T- and B-cell mitogens were restored to normal levels following vitamin C supplementation [198]. Although the human studies mentioned above are encouraging, it is apparent that more human intervention studies are needed to confirm these findings.

Recent research in wild-type and Gulo knockout mice indicated that parenteral administration of 200 mg/kg vitamin C modulated the immunosuppression of regulatory T-cells (Tregs) observed in sepsis [89]. Vitamin C administration enhanced Treg proliferation and inhibited the negative immunoregulation of Tregs by inhibiting the expression of specific transcription factors, antigens, and cytokines [89]. The mechanisms involved likely rely on the gene regulatory effects of vitamin C [79,89,199,200]. For example, recent research has implicated vitamin C in epigenetic regulation through its action as a cofactor for the iron-containing dioxygenases which hydroxylate methylated DNA and histones [22,201]. The ten-eleven translocation (TET) enzymes hydroxylate methylcytosine residues, which may act as epigenetic marks in their own right, and also facilitate removal of the methylated residues, an important process in epigenetic regulation [202]. Preliminary evidence indicates that vitamin C can regulate T-cell maturation via epigenetic mechanisms involving the TETs and histone demethylation [79,199,200]. It is likely that the cell signaling and gene regulatory functions of vitamin C, via regulation of transcription factors and epigenetic marks, play major roles in its immune-regulating functions.

### 3.6. Inflammatory Mediators

Cytokines are important cell signaling molecules secreted by a variety of immune cells, both innate and adaptive, in response to infection and inflammation [1]. They comprise a broad range of molecules, including chemokines, interferons (IFNs), ILs, lymphokines, and TNFs, which modulate both humoral and cell-based immune responses, and regulate the maturation, growth, and responsiveness of specific cell populations. Cytokines can elicit pro-inflammatory or anti-inflammatory responses, and vitamin C appears to modulate systemic and leukocyte-derived cytokines in a complex manner.

Incubation of vitamin C with peripheral blood lymphocytes decreased lipopolysaccharide (LPS)-induced generation of the pro-inflammatory cytokines TNF-α and IFN-γ, and increased anti-inflammatory IL-10 production, while having no effect on IL-1β levels [77]. Furthermore, in vitro addition of vitamin C to peripheral blood monocytes isolated from pneumonia patients decreased the generation of the pro-inflammatory cytokines TNF-α and IL-6 [86]. However, another study found that in vitro treatment of peripheral blood monocytes with vitamin C and/or vitamin E enhanced LPS-stimulated TNF-α generation, but did not affect IL-1β generation [87]. Furthermore, incubation of vitamin C with virus-infected human and murine fibroblasts enhanced generation of antiviral IFN [91,92,93]. Supplementation of healthy human volunteers with 1 g/day vitamin C (with and without vitamin E) was shown to enhance peripheral blood mononuclear cell-derived IL-10, IL-1, and TNF-α following stimulation with LPS [87,94]. Thus, the effect of vitamin C on cytokine generation appears to depend on the cell type and/or the inflammatory stimulant. Recent research has indicated that vitamin C treatment of microglia, resident myeloid-derived macrophages in the central nervous system, attenuates activation of the cells and synthesis of the pro-inflammatory cytokines TNF, IL-6, and IL-1β [90]. This is indicative of an anti-inflammatory phenotype.

Preclinical studies using Gluo knockout mice have highlighted the cytokine-modulating effects of vitamin C. Vitamin C-deficient Gulo knockout mice infected with influenza virus showed enhanced synthesis of the pro-inflammatory cytokines TNF-α and IL-1α/β in their lungs, and decreased production of the anti-viral cytokine IFN-α/β [88]. Administration of vitamin C to Gulo mice with polymicrobial peritonitis resulted in decreased synthesis of the pro-inflammatory cytokines TNF-α and IL-1β by isolated neutrophils [75]. Another study in septic Gulo mice administered 200 mg/kg parenteral vitamin C has shown decreased secretion of the inhibitory cytokines TGF-β and IL-10 by Tregs [89]. In this study, attenuated IL-4 secretion and augmented IFN-γ secretion was also observed, suggesting immune-modulating effects of vitamin C in sepsis. Overall, vitamin C appears to normalize cytokine generation, likely through its gene-regulating effects.

Histamine is an immune mediator produced by basophils, eosinophils, and mast cells during the immune response to pathogens and stress. Histamine stimulates vasodilation and increased capillary permeability, resulting in the classic allergic symptoms of runny nose and eyes. Studies using guinea pigs, a vitamin C-requiring animal model, have indicated that vitamin C depletion is associated with enhanced circulating histamine levels, and that supplementation of the animals with vitamin C resulted in decreased histamine levels [56,95,96,97,98]. Enhanced histamine generation was found to increase the utilization of vitamin C in these animals [96]. Consistent with the animal studies, human intervention studies with oral vitamin C (125 mg/day to 2 g/day) and intravenous vitamin C (7.5 g infusion) have reported decreased histamine levels [61,99,100,101], which was more apparent in patients with allergic compared with infectious diseases [101]. Although vitamin C has been proposed to ‘detoxify’ histamine [96,97], the precise mechanisms responsible for the in vivo decrease in histamine levels following vitamin C administration are currently unknown. Furthermore, effects of vitamin C supplementation on histamine levels are not observed in all studies [203].

## 4. Vitamin C Insufficiency Conditions

Numerous environmental and health conditions can have an impact on vitamin C status. In this section we discuss examples which also have a link with impaired immunity and increased susceptibility to infection. For example, exposure to air pollution containing oxidants, such as ozone and nitrogen dioxide, can upset the oxidant-antioxidant balance within the body and cause oxidative stress [204]. Oxidative stress can also occur if antioxidant defenses are impaired, which may be the case when vitamin C levels are insufficient [205]. Air pollution can damage respiratory tract lining fluid and increase the risk of respiratory disease, particularly in children and the elderly [204,206] who are at risk of both impaired immunity and vitamin C insufficiency [14,204]. Vitamin C is a free-radical scavenger that can scavenge superoxide and peroxyl radicals, hydrogen peroxide, hypochlorous acid, and oxidant air pollutants [207,208]. The antioxidant properties of vitamin C enable it to protect lung cells exposed to oxidants and oxidant-mediated damage caused by various pollutants, heavy metals, pesticides, and xenobiotics [204,209].

Tobacco smoke is an underestimated pollutant in many parts of the world. Both smokers and passive smokers have lower plasma and leukocyte vitamin C levels than non-smokers [10,210,211], partly due to increased oxidative stress and to both a lower intake and a higher metabolic turnover of vitamin C compared to non-smokers [10,211,212,213]. Mean serum concentrations of vitamin C in adults who smoke have been found to be one-third lower than those of non-smokers, and it has been recommended that smokers should consume an additional 35 mg/day of vitamin C to ensure there is sufficient ascorbic acid to repair oxidant damage [10,14]. Vitamin C levels are also lower in children and adolescents exposed to environmental tobacco smoke [214]. Research in vitamin C-deficient guinea pigs exposed to tobacco smoke has indicated that vitamin C can protect against protein damage and lipid peroxidation [213,215]. In passive smokers exposed to environmental tobacco smoke, vitamin C supplementation significantly reduced plasma F_2_-isoprostane concentrations, a measure of oxidative stress [216]. Tobacco use increases susceptibility to bacterial and viral infections [217,218], in which vitamin C may play a role. For example, in a population-based study the risk of developing obstructive airways disease was significantly higher in those with the lowest plasma vitamin C concentrations (26 µmol/L) compared to never smokers, a risk that decreased with increasing vitamin C concentration [219].

Individuals with diabetes are at greater risk of common infections, including influenza, pneumonia, and foot infections, which are associated with increased morbidity and mortality [220,221]. Several immune-related changes are observed in obesity that contribute towards the development of type 2 diabetes. A major factor is persistent low-grade inflammation of adipose tissue in obese subjects, which plays a role in the progression to insulin resistance and type 2 diabetes, and which is not present in the adipose tissue of lean subjects [222,223]. The adipose tissue is infiltrated by pro-inflammatory macrophages and T-cells, leading to the accumulation of pro-inflammatory cytokines such as interleukins and TNF-α [224,225]. A decrease in plasma vitamin C levels has been observed in studies of type 2 diabetes [18,226], and a major cause of increased need for vitamin C in type 2 diabetes is thought to be the high level of oxidative stress caused by hyperglycemia [10,227,228]. Inverse correlations have been reported between plasma vitamin C concentrations and the risk of diabetes, hemoglobin A1c concentrations (an index of glucose tolerance), fasting and postprandial blood glucose, and oxidative stress [219,229,230,231,232]. Meta-analysis of interventional studies has indicted that supplementation with vitamin C can improve glycemic control in type 2 diabetes [233].

Elderly people are particularly susceptible to infections due to immunosenescence and decreased immune cell function [234]. For example, common viral infections such as respiratory illnesses, that are usually self-limiting in healthy young people, can lead to the development of complications such as pneumonia, resulting in increased morbidity and mortality in elderly people. A lower mean vitamin C status has been observed in free-living or institutionalized elderly people, indicated by lowered plasma and leukocyte concentrations [10,235,236], which is of concern because low vitamin C concentrations (<17 µmol/L) in older people (aged 75–82 years) are strongly predictive of all-cause mortality [237]. Acute and chronic diseases that are prevalent in this age group may also play an important part in the reduction of vitamin C reserves [238,239,240]. Institutionalization in particular is an aggravating factor in this age group, resulting in even lower plasma vitamin C levels than in non-institutionalized elderly people. It is noteworthy that elderly hospitalized patients with acute respiratory infections have been shown to fare significantly better with vitamin C supplementation than those not receiving the vitamin [241]. Decreased immunological surveillance in individuals older than 60 years also results in greater risk of cancer, and patients with cancer, particularly those undergoing cancer treatments, have compromised immune systems, decreased vitamin C status, and enhanced risk of developing sepsis [242,243]. Hospitalized patients, in general, have lower vitamin C status than the general population [244].

## 5. Vitamin C and Infection

A major symptom of the vitamin C deficiency disease scurvy is the marked susceptibility to infections, particularly of the respiratory tract, with pneumonia being one of the most frequent complications of scurvy and a major cause of death [7]. Patients with acute respiratory infections, such as pulmonary tuberculosis and pneumonia, have decreased plasma vitamin C concentrations relative to control subjects [245]. Administration of vitamin C to patients with acute respiratory infections returns their plasma vitamin C levels to normal and ameliorates the severity of the respiratory symptoms [246]. Cases of acute lung infections have shown rapid clearance of chest X-rays following administration of intravenous vitamin C [247,248]. This vitamin C-dependent clearance of neutrophils from infected lungs could conceivably be due to enhanced apoptosis and subsequent phagocytosis and clearance of the spent neutrophils by macrophages [73]. Pre-clinical studies of animals with sepsis-induced lung injury have indicated that vitamin C administration can increase alveolar fluid clearance, enhance bronchoalveolar epithelial barrier function, and attenuate sequestration of neutrophils [74], all essential factors for normal lung function.

Meta-analysis has indicated that vitamin C supplementation with doses of 200 mg or more daily is effective in ameliorating the severity and duration of the common cold, and the incidence of the common cold if also exposed to physical stress [249]. Supplementation of individuals who had an inadequate vitamin C status (i.e., <45 μmol/L) also decreased the incidence of the common cold [203]. Surprisingly, few studies have assessed vitamin C status during the common cold [250]. Significant decreases in both leukocyte vitamin C levels, and urinary excretion of the vitamin, have been reported to occur during common cold episodes, with levels returning to normal following the infection [251,252,253,254]. These changes indicate that vitamin C is utilized during the common cold infection. Administration of gram doses of vitamin C during the common cold episode ameliorated the decline in leukocyte vitamin C levels, suggesting that administration of vitamin C may be beneficial for the recovery process [251].

Beneficial effects of vitamin C on recovery have been noted in pneumonia. In elderly people hospitalized because of pneumonia, who were determined to have very low vitamin C levels, administration of vitamin C reduced the respiratory symptom score in the more severe patients [246]. In other pneumonia patients, low-dose vitamin C (0.25–0.8 g/day) reduced the hospital stay by 19% compared with no vitamin C supplementation, whereas the higher-dose group (0.5–1.6 g/day) reduced the duration by 36% [255]. There was also a positive effect on the normalization of chest X-ray, temperature, and erythrocyte sedimentation rate [255]. Since prophylactic vitamin C administration also appears to decrease the risk of developing more serious respiratory infections, such as pneumonia [256], it is likely that the low vitamin C levels observed during respiratory infections are both a cause and a consequence of the disease.

## 6. Conclusions

Overall, vitamin C appears to exert a multitude of beneficial effects on cellular functions of both the innate and adaptive immune system. Although vitamin C is a potent antioxidant protecting the body against endogenous and exogenous oxidative challenges, it is likely that its action as a cofactor for numerous biosynthetic and gene regulatory enzymes plays a key role in its immune-modulating effects. Vitamin C stimulates neutrophil migration to the site of infection, enhances phagocytosis and oxidant generation, and microbial killing. At the same time, it protects host tissue from excessive damage by enhancing neutrophil apoptosis and clearance by macrophages, and decreasing neutrophil necrosis and NETosis. Thus, it is apparent that vitamin C is necessary for the immune system to mount and sustain an adequate response against pathogens, whilst avoiding excessive damage to the host.

Vitamin C appears to be able to both prevent and treat respiratory and systemic infections by enhancing various immune cell functions. Prophylactic prevention of infection requires dietary vitamin C intakes that provide at least adequate, if not saturating plasma levels (i.e., 100–200 mg/day), which optimize cell and tissue levels. In contrast, treatment of established infections requires significantly higher (gram) doses of the vitamin to compensate for the increased metabolic demand.

Epidemiological studies indicate that hypovitaminosis C is still relatively common in Western populations, and vitamin C deficiency is the fourth leading nutrient deficiency in the United States. Reasons include reduced intake combined with limited body stores. Increased needs occur due to pollution and smoking, fighting infections, and diseases with oxidative and inflammatory components, e.g., type 2 diabetes, etc. Ensuring adequate intake of vitamin C through the diet or via supplementation, especially in groups such as the elderly or in individuals exposed to risk factors for vitamin C insufficiency, is required for proper immune function and resistance to infections.

## Figures and Tables

**Table 1:** Role of vitamin C in immune defense.

Immune System	Function of Vitamin C	Refs.
Epithelial barriers	Enhances collagen synthesis and stabilization	[30,31,32,33,34,35]
	Protects against ROS-induced damage ^1^	[36,37,38,39,40]
	Enhances keratinocyte differentiation and lipid synthesis	[41,42,43,44,45]
	Enhances fibroblast proliferation and migration	[46,47]
	Shortens time to wound healing in patients	[48,49]
Phagocytes (neutrophils, macrophages)	Acts as an antioxidant/electron donor	[50,51,52,53]
	Enhances motility/chemotaxis	[54,55,56,57,58,59,60,61,62,63]
	Enhances phagocytosis and ROS generation	[64,65,66,67,68,69,70,71]
	Enhances microbial killing	[54,55,57,58,70,72]
	Facilitates apoptosis and clearance	[71,73,74]
	Decreases necrosis/NETosis	[73,75]
B- and T-lymphocytes	Enhances differentiation and proliferation	[62,63,76,77,78,79,80,81,82]
	Enhances antibody levels	[78,83,84,85]
Inflammatory mediators	Modulates cytokine production	[75,77,86,87,88,89,90,91,92,93,94]
	Decreases histamine levels	[56,61,95,96,97,98,99,100,101]

^1^ ROS, reactive oxygen species; NET, neutrophil extracellular trap. Note that many of these studies comprised marginal or deficient vitamin C status at baseline. Supplementation in situations of adequate vitamin C status may not have comparable effects.
